# Supplementary material for: Correlation between B7-H4 and Survival of Non-Small-Cell Lung Cancer Patients Treated with Nivolumab
Source: J Clin Med. 2019 Oct 1;8(10):1566. doi: 10.3390/jcm8101566 (PMC6832616; doi:10.3390/jcm8101566)
Supplement: Supplementary file 1 [file jcm-08-01566-s001.pdf]

Supplementary Tables

Supplementary Table S1. Distribution of the biomarker expression among the samples collected from each patient within the Nivolumab Cohort. For each marker the positive tumor cells (%) and the intensity were reported. The percentage of stained tumor cells were arbitrarily graded as follows: <1% (negative), 1%–9% (low expression), 10%–49% (moderate expression), ≥50% (high expression), whereas the intensity of staining was categorized as follows: absent (0), weak (1+), moderate (2+), and strong (3+). ND: not determined.

PATIENT ID	PD-L1		PD-L2		PD-1		B7-H3		B7-H4	
	% Tumor Cells	Intensity	% Tumor Cells	Intensity	% Tumor Cells	Intensity	% Tumor Cells	Intensity	% Tumor Cells	Intensity
**1**	<1%	0	10%–49%	2+	<1%	0	<1%	0	<1%	0
**2**	<1%	0	<1%	0	1%–9%	1+	<1%	0	1%–9%	1+
**3**	<1%	0	<1%	0	1%–9%	2+	<1%	0	≥50%	2+
**4**	<1%	0	<1%	0	10%–49%	1+	<1%	0	<1%	0
**5**	<1%	0	<1%	0	≥50%	3+	<1%	0	<1%	0
**6**	<1%	0	<1%	0	≥50%	3+	10%–49%	1+	≥50%	3+
**7**	<1%	0	<1%	0	≥50%	2+	<1%	0	≥50%	1+
**8**	<1%	0	<1%	0	<1%	0	<1%	0	<1%	0
**9**	<1%	0	<1%	0	<1%	0	10%–49%	1+	<1%	0
**10**	<1%	0	<1%	0	<1%	0	<1%	0	<1%	0
**11**	1%–9%	2+	<1%	0	1%–9%	1+	<1%	0	1%–9%	1+
**12**	<1%	0	<1%	0	≥50%	3+	1%–9%	1+	<1%	0
**13**	<1%	0	<1%	0	<1%	0	<1%	0	<1%	0
**14**	<1%	0	<1%	0	1%–9%	1+	<1%	0	<1%	0
**15**	<1%	0	<1%	0	≥50%	0	<1%	0	<1%	0
**16**	≥50%	3+	<1%	0	≥50%	2+	<1%	0	≥50%	1+
**17**	<1%	0	<1%	0	10%–49%	2+	<1%	0	≥50%	3+
**18**	<1%	0	<1%	0	≥50%	2+	<1%	0	10%–49%	1+
**19**	<1%	0	<1%	0	≥50%	1+	<1%	0	<1%	0
**20**	<1%	0	<1%	0	ND	ND	≥50%	3+	1%–9%	1+
**21**	<1%	0	<1%	0	10%–49%	2+	10%–49%	1+	10%–49%	1+
**22**	<1%	0	<1%	0	1%–9%	1+	<1%	0	≥50%	1+
**23**	<1%	0	1%–9%	1+	10%–49%	2+	<1%	0	<1%	0
**24**	<1%	0	<1%	0	<1%	0	<1%	0	<1%	0
**25**	<1%	0	10%–49%	1+	<1%	0	<1%	0	<1%	0
**26**	1%–9%	1+	<1%	0	≥50%	1+	<1%	0	<1%	0
**27**	10%–49%	2+	10%–49%	1+	≥50%	2+	<1%	0	10%–49%	1+
**28**	<1%	0	<1%	0	<1%	0	<1%	0	<1%	0

**29**	<1%	0	<1%	0	≥50%	2+	<1%	0	<1%	0
**30**	<1%	0	<1%	0	≥50%	1+	<1%	0	<1%	0
**31**	1%–9%	1+	<1%	0	≥50%	3+	<1%	0	<1%	0
**32**	<1%	0	<1%	0	<1%	0	<1%	0	<1%	0
**33**	<1%	0	<1%	0	<1%	0	<1%	0	≥50%	2+
**34**	<1%	0	<1%	0	1%–9%	1+	<1%	0	<1%	0
**35**	<1%	0	<1%	0	1%–9%	1+	<1%	0	<1%	0
**36**	1–9%	1+	<1%	0	≥50%	3+	<1%	0	<1%	0
**37**	<1%	0	1%–9%	1+	1%–9%	1+	<1%	0	<1%	0
**38**	<1%	0	10%–49%	1+	<1%	0	<1%	0	<1%	0
**39**	10%–49%	3+	<1%	0	10%–49%	1+	<1%	0	≥50%	2+
**40**	<1%	0	<1%	0	ND	ND	<1%	0	<1%	0
**41**	<1%	0	<1%	0	<1%	0	1%–9%	1+	1–9%	1+
**42**	ND	ND	<1%	0	≥50%	2+	<1%	0	<1%	0
**43**	<1%	0	<1%	0	≥50%	2+	<1%	0	1–9%	1+
**44**	<1%	0	<1%	0	<1%	0	<1%	0	<1%	0
**45**	<1%	0	≥50%	2+	≥50%	3+	<1%	0	<1%	0
**46**	<1%	0	10%–49%	1+	≥50%	1+	<1%	0	10%–49%	1+

Supplementary Table S2. Overall outcome data in the Nivolumab Cohort. Among the patients from the Nivolumab Cohort, one was considered evaluable for irRC, but not for RECIST, based on measurable lesions; one additional patient was considered not evaluable as he/she discontinued treatment and did not undergo further CT scans after baseline; both patients were followed for overall survival.

NIVOLUMAB COHORT N = 46		
	**Recist**	**irRC**
**Evaluable patients for PFS**	N = 44	N = 45
**Median PFS (95% CI)**	1.9 months (1.7–2.2)	1.9 months (1.7–2.9)
**ORR**	14.0%	13.6%
**DCR**	27.9%	38.6%
**Median OS (95% CI)**	8.9 months (4.4–12.2)	

Among the patients who were evaluable for PFS, one missed the first scheduled response assessment, although subsequent scans were available for PFS; hence, this patient was excluded from ORR and DCR analysis.

Supplementary Table S3. Distribution of the biomarkers expression among the samples collected from each patient within the Chemotherapy Cohort. For each marker the positive tumor cells (%) and the intensity were reported. The percentage of stained tumor cells were arbitrarily graded as follows: <1% (negative), 1%–9% (low expression), 10%–49% (moderate expression), ≥50% (high expression), whereas the intensity of staining was categorized as follows: absent (0), weak (1+), moderate (2+), and strong (3+).

PATIENT ID	PD-L1		B7-H4	
	**% Tumor Cells**	**Intensity**	**% Tumor Cells**	**Intensity**
**1**	<1%	0	10%–49%	2+
**2**	<1%	0	≥50%	3+
**3**	<1%	0	≥50%	2+
**4**	<1%	0	≥50%	1+
**5**	1%–9%	1+	1–9%	1+
**6**	<1%	0	<1%	0
**7**	<1%	0	<1%	0
**8**	<1%	0	<1%	0
**9**	<1%	0	1%–9%	1+
**10**	<1%	0	≥50%	3+
**11**	<1%	0	≥50%	2+
**12**	<1%	0	<1%	0
**13**	10%–49%	1+	<1%	0
**14**	1–9%	1+	≥50%	2+
**15**	<1%	0	10%–49%	2+
**16**	<1%	0	≥50%	1+
**17**	<1%	0	<1%	0
**18**	<1%	0	1–9%	3+
**19**	<1%	0	10%–49%	2+
**20**	1%–9%	1+	<1%	0
**21**	<1%	0	<1%	0
**22**	<1%	0	≥50%	2+
**23**	<1%	0	<1%	0
**24**	<1%	0	<1%	0
**25**	1%–9%	1+	≥50%	2+
**26**	1%–9%	1+	<1%	0
**27**	<1%	0	<1%	0

Supplementary Table S4. Global outcome data in the Chemotherapy Cohort.

CHEMOTHERAPY COHORT N = 27	
**Evaluable patients for PFS**	N = 27
**Median PFS (95% CI)**	3.3 months (2.4–6.7)
**ORR**	18.5%
**DCR**	74.1%
**Median OS (95% CI)**	8.3 months (4.3–13.2)

Supplementary Figures

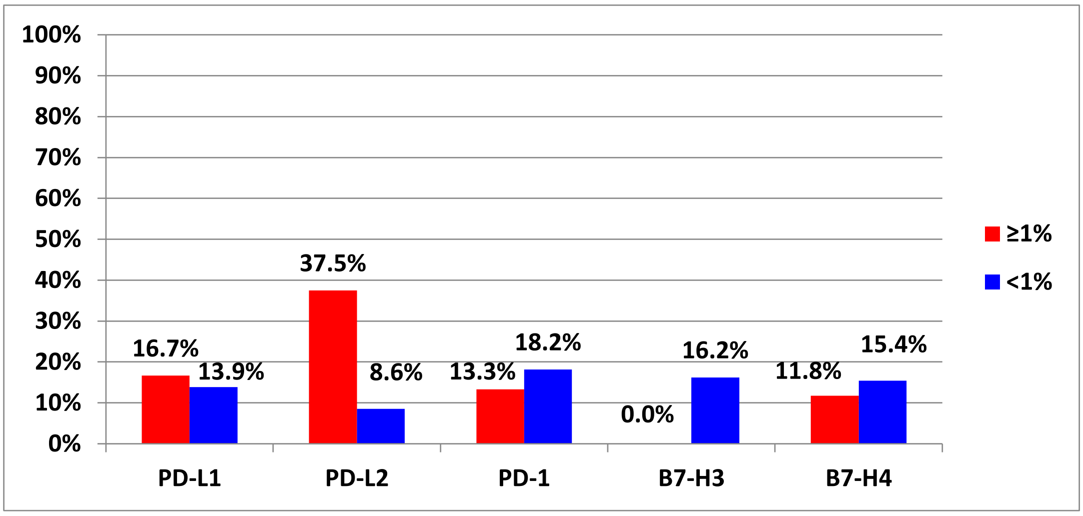

Supplementary Figure S1. Correlation between RECIST-ORR and biomarkers expression in the Nivolumab cohort. No statistically significant interaction was observed between each biomarker and ORR, although the correlation between PD-L2 expression and RECIST-ORR was close to significance (*p*-value = 0.067).

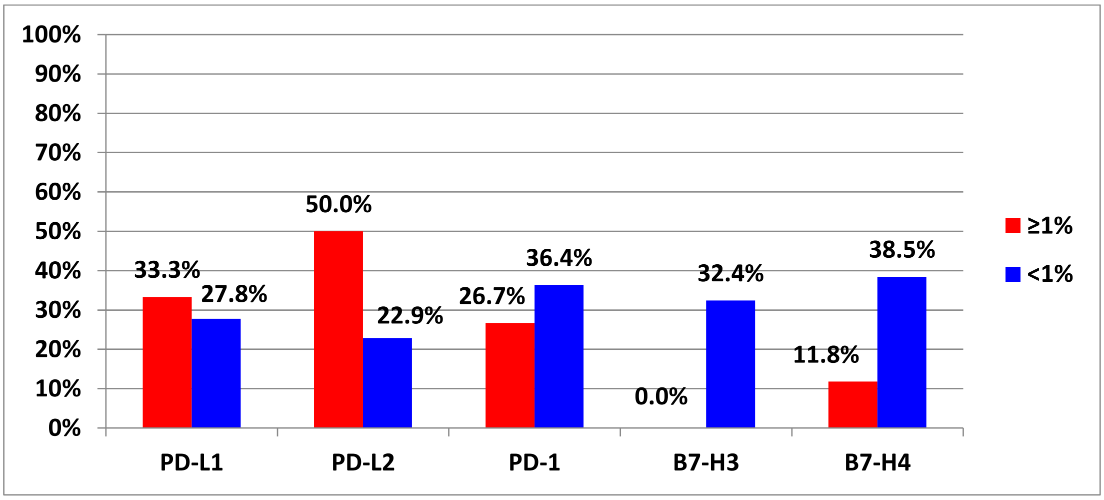

Supplementary Figure S2. Correlation between RECIST-DCR and biomarkers expression in the Nivolumab cohort. No statistically significant interaction was observed between each biomarker and DCR, although the correlation between B7-H4 expression and RECIST-DCR was close to significance (*p*-value = 0.085).

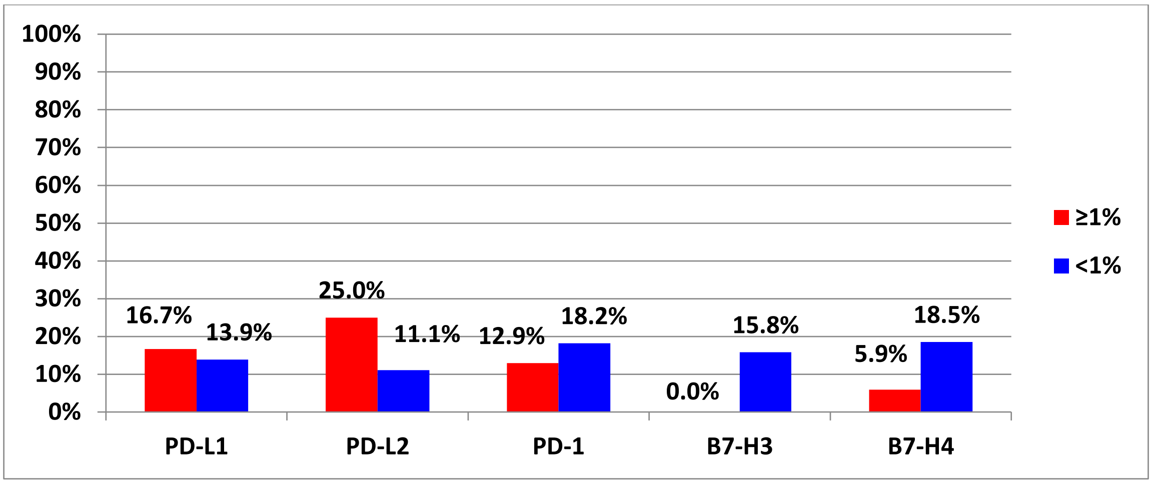

Supplementary Figure S3. Correlation between immune-related response criteria (irRC)-ORR and biomarkers expression in the Nivolumab cohort. irRC-ORR according to biomarkers expression at IHC in the Nivolumab Cohort. No statistically significant interaction was observed between each biomarker and ORR.

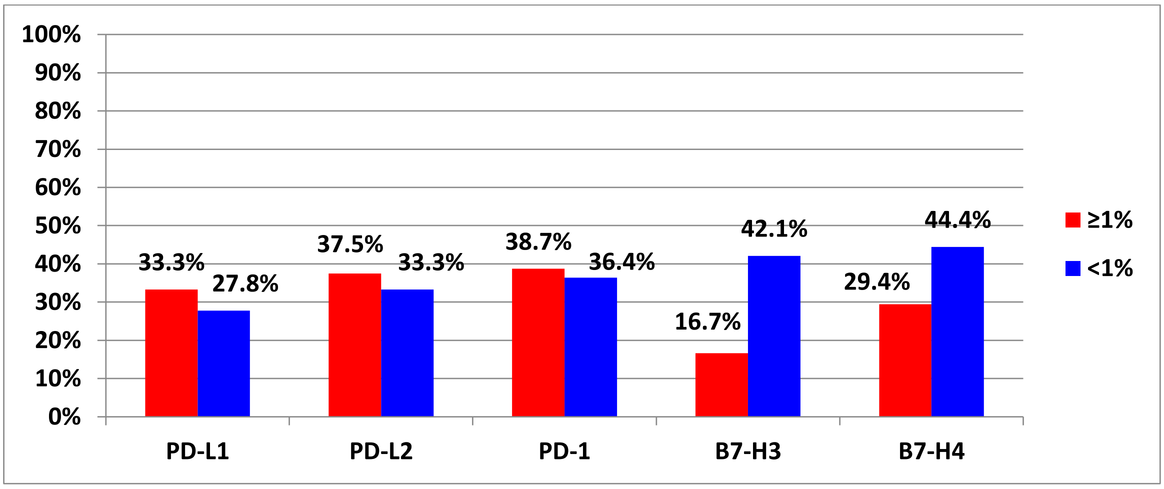

Supplementary Figure S4. Correlation between irRC-DCR and biomarkers expression in the Nivolumab cohort. No significant interaction was observed between each biomarker and DCR.

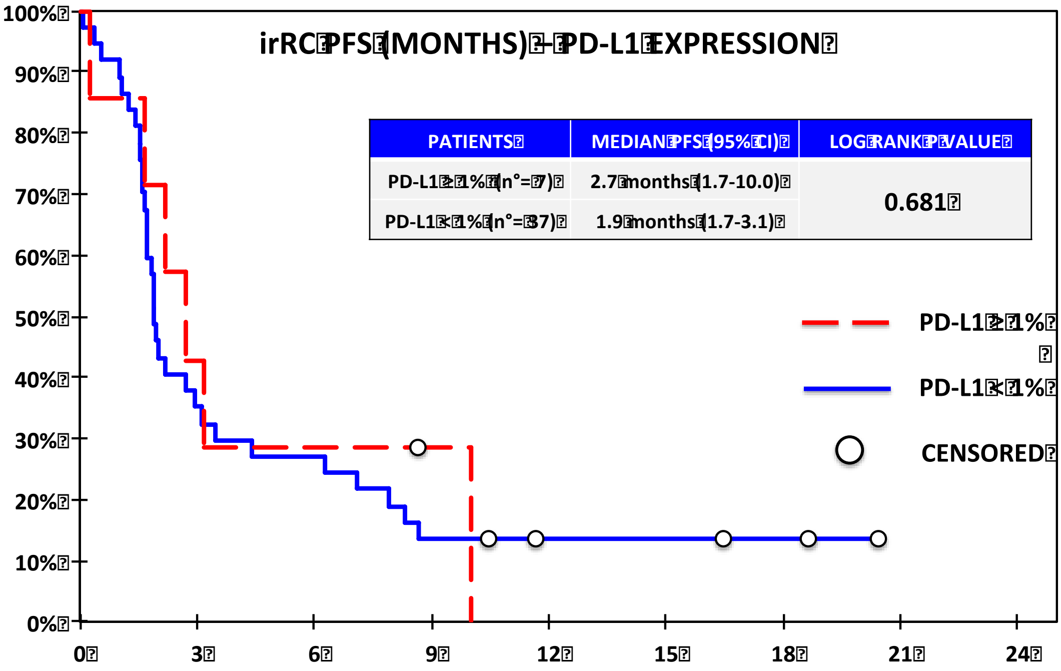

N° at risk

≥1	7	3	2	1	0			
<1	37	14	11	5	3	3	3	0

Supplementary Figure S5. irRC-PFS based on the expression of PD-L1 defined as ≥1% vs. <1% in the Nivolumab Cohort. No significant difference was observed on the basis of PD-L1 expression.

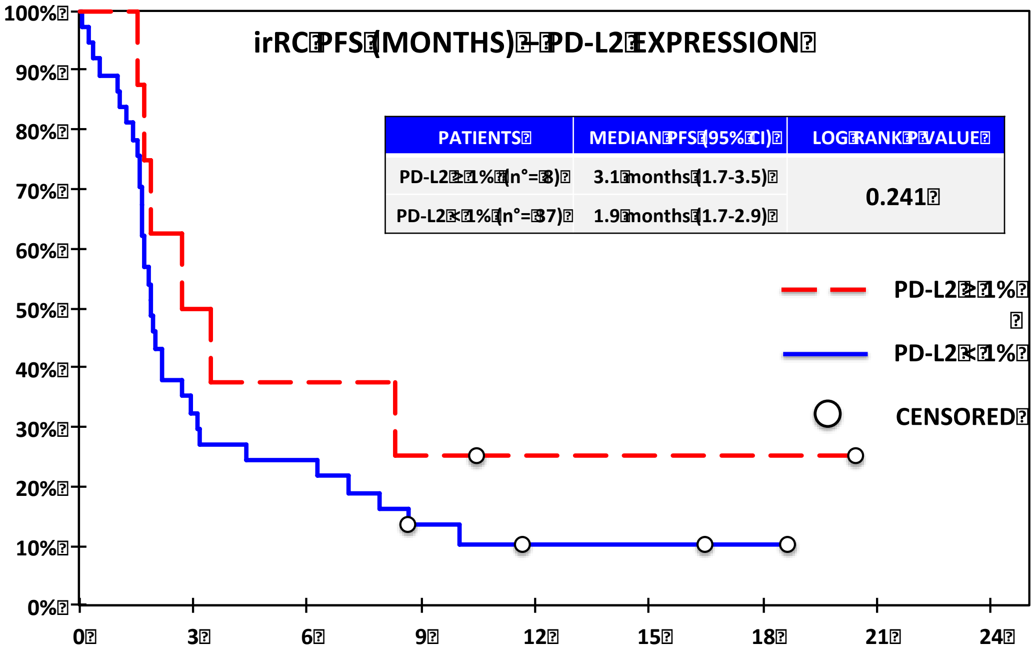

N° at risk

≥1	8	4	3	2	1	1	1	0
<1	37	12	9	4	2	2	1	0

Supplementary Figure 6. irRC-PFS based on the expression of PD-L2 defined as ≥1% vs. <1% in the Nivolumab Cohort. No significant difference was observed on the basis of PD-L2 expression.

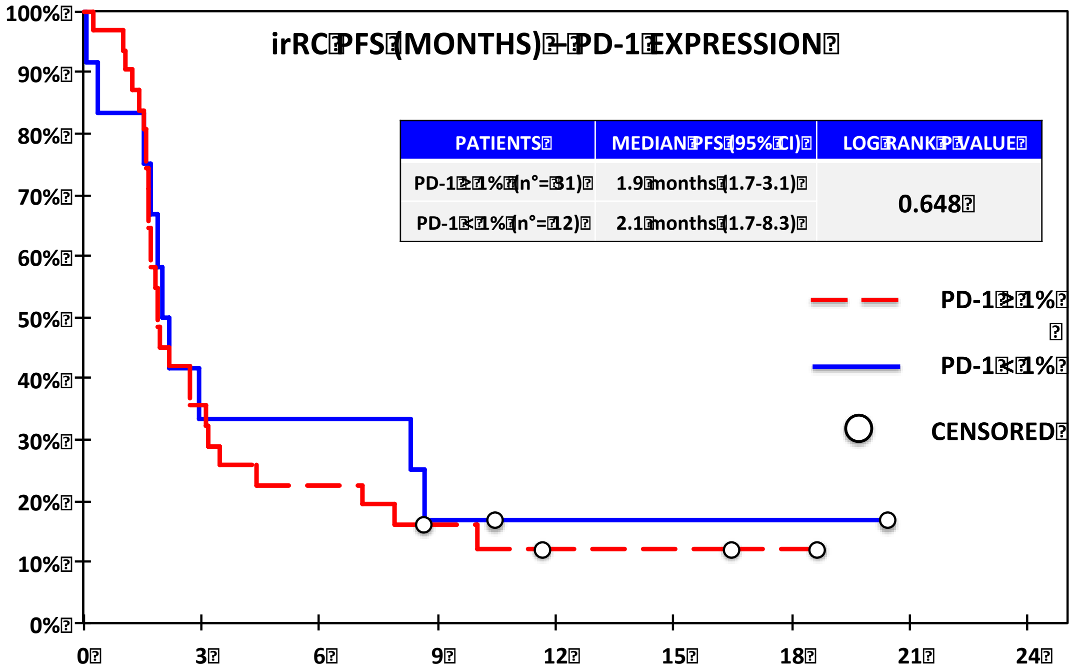

PATIENTS	MEDIAN PFS (95% CI)	LOG RANK P VALUE
PD-1 ≥ 1% (n° = 31)	1.9 months (1.7-3.1)	0.648
PD-1 < 1% (n° = 12)	2.1 months (1.7-8.3)	

N° at risk

≥1	31	11	7	4	2	2	1	0
<1	12	4	4	2	1	1	1	0

Supplementary Figure S7. irRC-PFS based on the expression of PD-1 defined as ≥1% vs. <1% in the Nivolumab Cohort. No significant difference was observed on the basis of PD-1 expression.

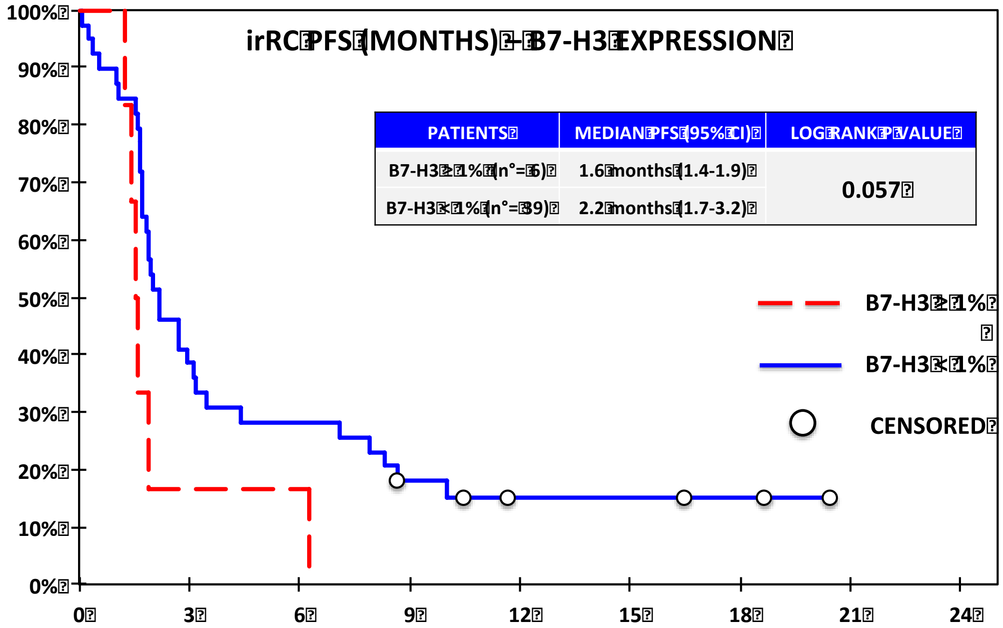

PATIENTS	MEDIAN PFS (95% CI)	LOG RANK P VALUE
B7-H3 ≥ 1% (n° = 6)	1.6 months (1.4-1.9)	0.057
B7-H3 < 1% (n° = 39)	2.2 months (1.7-3.2)	

N° at risk

≥1	6	1	1	0				
<1	39	15	11	6	3	3	2	0

Supplementary Figure S8. irRC-PFS based on the expression of B7-H3 defined as ≥1% vs. <1% in the Nivolumab Cohort. irRC-PFS difference based on B7-H3 expression fell short of statistical significance (p-value = 0.057); while this result should be considered with caution due to the fact that only six out of 44 patients expressed B7-H3, the rapid progression of B7-H3-expressing patients was noteworthy.

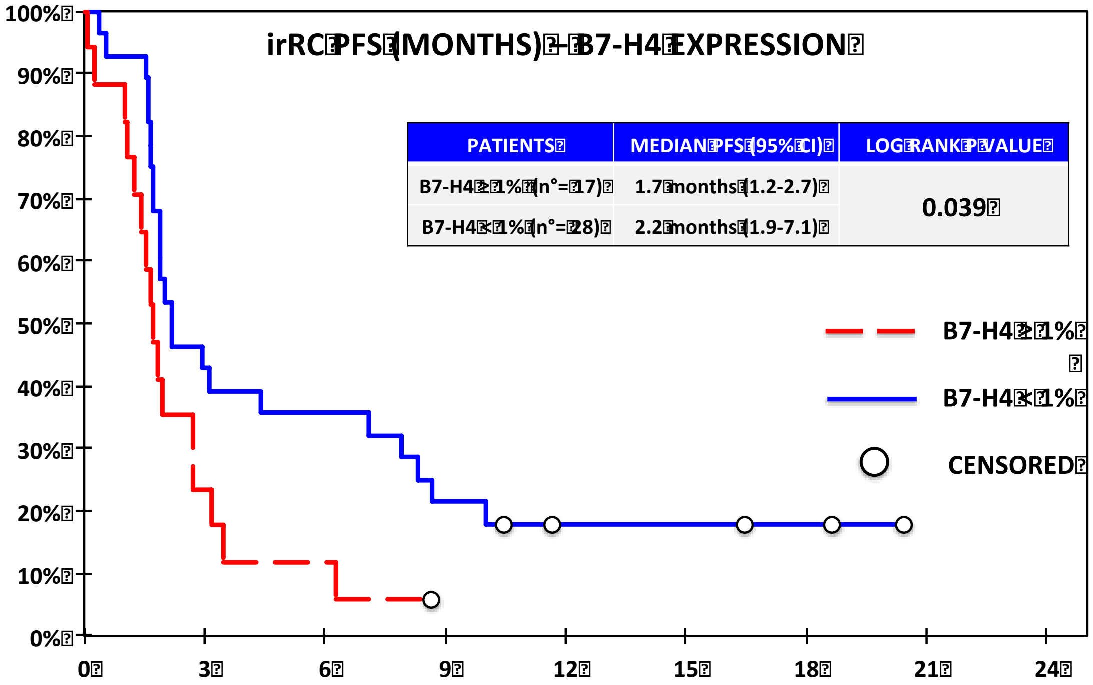

N° at risk

≥1	17	4	2	0				
<1	28	12	10	6	3	3	2	0

Supplementary Figure S9. irRC-PFS based on the expression of B7-H4 defined as ≥1% vs. <1% in the Nivolumab Cohort. The expression of B7-H4 was significantly associated with shorter PFS.

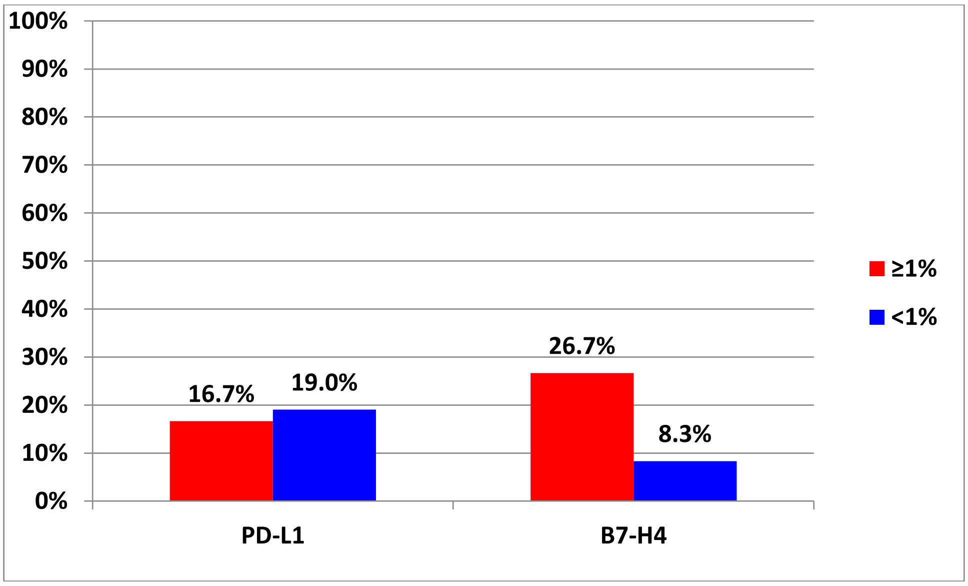

Supplementary Figure S10. Correlation between RECIST-ORR and biomarkers expression in the Chemotherapy cohort. No significant interaction was observed between each biomarker and ORR.

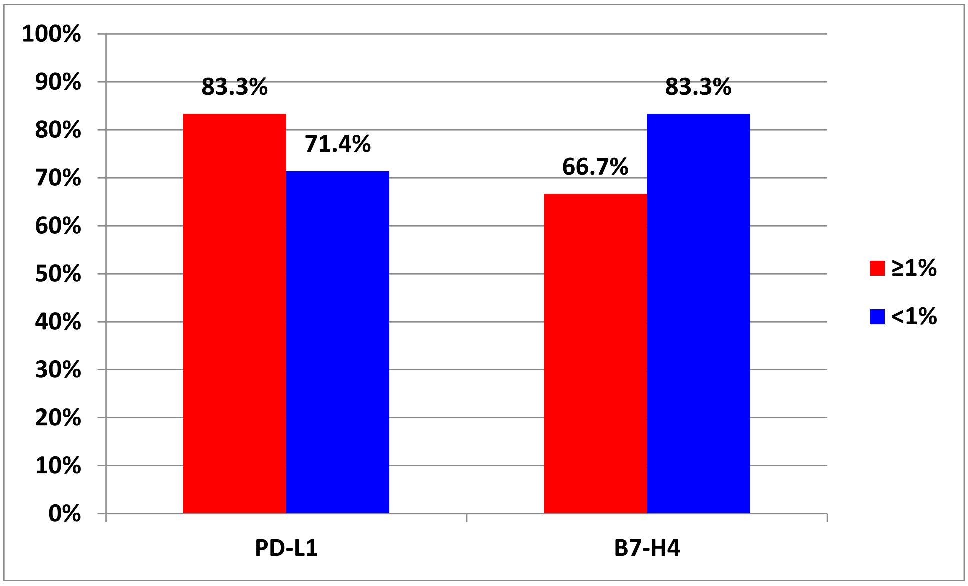

Supplementary Figure S11. Correlation between RECIST-DCR and biomarkers expression in the Chemotherapy cohort. No significant interaction was observed between each biomarker and DCR.
